# Antioxidant and Anti-Inflammatory Activities of Thai Traditional Hand and Foot Soaking Formulary and Its Bioactive Compounds

**DOI:** 10.3390/pharmaceutics17070907

**Published:** 2025-07-13

**Authors:** Jaenjira Angsusing, Weerasak Samee, Supachoke Mangmool, Usma Dortae, Pranot Keawthip, Surakameth Mahasirimongkol, Somsak Kreechai, Kulthanit Wanaratna, Chuda Chittasupho, Nopparut Toolmal

**Affiliations:** 1 Department of Thai Traditional and Alternative Medicine, Ministry of Public Health, Nonthaburi 11000, Thailand; jaenjira.angsusing@hotmail.com (J.A.); usma.do@dtam.mail.go.th (U.D.); pranot.ka@dtam.mail.go.th (P.K.); surakameth.m@dmsc.mail.go.th (S.M.); somsak.kreechaii@gmail.com (S.K.); kulthanitw@gmail.com (K.W.); 2 Faculty of Pharmacy, Srinakharinwirot University, Nakhon Nayok 26120, Thailand; weerasak@g.swu.ac.th; 3 Faculty of Pharmacy, Chiang Mai University, Chiang Mai 50200, Thailand; supachoke.man@cmu.ac.th

**Keywords:** Thai traditional medicine, hand and foot soaking formulary, curcumin, antioxidant activity, anti-inflammatory activity

## Abstract

**Background/Objectives**: This study aimed to investigate the antioxidant and anti-inflammatory properties of a Hand and Foot Soaking Formulary composed of ten medicinal plants, with curcumin as a major bioactive marker, to provide scientific validation for its traditional use. **Methods:** The formulation was evaluated for total phenolic and flavonoid contents, with curcumin quantified using HPLC. Antioxidant activity was assessed using DPPH, ABTS, and FRAP assays. Cytotoxicity was evaluated in RAW264.7 cells using the MTT assay. Anti-inflammatory activity was determined by measuring nitric oxide (NO), PGE_2_, TNF-α, IL-1β, and IL-6 levels in LPS-stimulated RAW264.7 macrophages using ELISA. **Results**: The Hand and Foot Soaking Formulary exhibited promising antioxidant and anti-inflammatory properties, consistent with its traditional use. Phytochemical analysis confirmed the presence of bioactive compounds, with measurable levels of total phenolics, flavonoids, and significant curcumin content. Antioxidant activity was demonstrated through free radical scavenging and ferric-reducing assays, while cytotoxicity testing in RAW264.7 macrophages indicated low toxicity (IC_50_ = 48.61 ± 3.80 µg/mL). The formulary significantly reduced LPS-induced nitric oxide, PGE_2_, TNF-α, IL-1β, and IL-6 production. These effects were comparable to turmeric extract and curcumin, though curcumin displayed higher potency. **Conclusions**: The Hand and Foot Soaking Formulary demonstrates antioxidant and anti-inflammatory properties in vitro, supporting its traditional use. Its polyherbal composition may offer synergistic effects and holds promise as a safe, natural topical remedy.

## 1. Introduction

Thai Traditional Medicine (TTM) is a holistic healthcare system rooted in Thai culture, blending herbal medicine, massage, and spiritual healing. TTM is now integrated into Thailand’s healthcare system, supported by regulation, formal education, and inclusion in universal health coverage. Scientific studies increasingly validate their practices, though challenges remain in standardizing complex remedies and preserving oral traditions. TTM has long incorporated herbal therapies into holistic health practices, with hand and foot soaking emerging as a prominent method for promoting relaxation, improving circulation, and alleviating pain and inflammation. This practice combines the therapeutic warmth of water immersion with the transdermal absorption of bioactive herbal compounds, offering both physiological and psychological benefits. Commonly used in Thai wellness centers, community hospitals, and elder care, herbal soaking is frequently prescribed for individuals experiencing joint stiffness, muscle fatigue, cold extremities, or inflammatory conditions.

Recent clinical observations have highlighted the growing interest in integrating such traditional interventions into modern healthcare. A study by Huang et al. demonstrated that both high- and low-dose Tangbi Waixi Decoction foot baths significantly improved neuropathic symptoms and nerve conduction in patients with diabetic peripheral neuropathy [1,2]. These findings underscore the potential of herbal soaking as a non-invasive, complementary therapy that can enhance quality of life, particularly in palliative and supportive care settings.

The formulation examined in this study, referred to as the Hand and Foot Soaking Formulary, is derived from classical Thai medicinal recipes. It includes ten herbs, *Curcuma longa* L. (20% *w*/*w*), *Piper retrofractum* Vahl (10%), *Cymbopogon citratus* (DC.) Stapf (10%), *Capparis micracantha* DC. (10%), *Clerodendrum indicum* (L.) Kuntze (10%), *Harrisonia perforata* (Blanco) Merr. (10%), *Tiliacora triandra* (Colebr.) Diels (10%), *Ficus racemosa* L. (10%), *Citrus hystrix* DC. (10%), and *Cinnamomum camphora* (L.) J.Presl (1%), each contributing specific parts traditionally used to reduce swelling, enhance peripheral blood flow, soothe sore muscles, and relieve joint pain. Notably, *C. longa* L. is widely recognized for its anti-inflammatory and analgesic properties, which are attributed to bioactive compounds such as curcuminoids, phenolics, and flavonoids [3,4]. However, the therapeutic efficacy of polyherbal formulations often extends beyond a single active compound. It is vital to acknowledge other herbs in the formulation for anti-inflammation and antioxidant activity. For example, piperine from *Piper retrofractum* [5], citral from *Cymbopogon citratus* [6], and naringin from *Citrus hystrix* [7] are known to suppress NF-κB activation and reduce pro-inflammatory cytokine release. These combined actions may contribute to synergistic effects resulting in an enhanced therapeutic outcome. Although curcumin has a lot of health benefits, poor bioavailability is the main challenge of the substance. However, combining other herbal ingredients was found to have improved curcumin bioavailability, enhancing antioxidant and anti-inflammatory synergistic effects, such as black pepper [8]. Moreover, mixing different polyphenols containing herbal components may overcome the bioavailability of natural compounds [9]. Similar synergistic findings were found in polyherbal-containing turmeric with *Zingiber officinale* Roscoe, *Cinnamomum verum* J.Presl, and *Capsicum annuum* L. for analgesic and anti-inflammatory effects [10]. Nevertheless, the anti-inflammatory properties of the Hand and Foot Soaking Formulary are not fully understood. The effects of the extract and some active compounds on the inhibition of LPS-induced upregulation of inflammatory mediators in macrophages have not been defined.

While such multi-herb formulations are frequently used in TTM practice, their chemical composition and biological activities remain uncharacterized, and they are compared to isolated compounds such as curcumin, which is the principal active constituent of turmeric (*C. longa*). Curcumin has been extensively studied for its potent antioxidant, anti-inflammatory, and anti-proliferative effects, making it a valuable reference standard for comparing the bioactivity of complex herbal mixtures [11,12].

Therefore, this study aimed to comprehensively evaluate the Hand and Foot Soaking Formulary by determining its total phenolic and flavonoid contents using validated colorimetric assays; quantifying its curcumin concentration via high-performance liquid chromatography (HPLC); assessing its antioxidant capacity using DPPH, ABTS, and FRAP assays; investigating cytotoxicity on RAW264.7 cells using the MTT assay; and exploring its anti-inflammatory effects in LPS-stimulated RAW264.7 macrophages by quantifying nitric oxide and key inflammatory cytokines (PGE_2_, TNF-α, IL-1β, IL-6) levels through ELISA. The LPS-stimulated RAW264.7 macrophage model is a widely used in vitro model for investigating anti-inflammatory activity, specifically the inhibition of inducible nitric oxide synthase (iNOS) and other inflammatory pathways [13]. Turmeric extract and pure curcumin were included as comparators to contextualize the pharmacological activity of the formulation.

We hypothesized that the Hand and Foot Soaking Formulary, due to its multi-herb composition that is rich in phenolic and curcuminoid compounds, would demonstrate significant antioxidant and anti-inflammatory activities, comparable to or greater than those of turmeric extract and curcumin, while maintaining low cytotoxicity, thereby supporting its potential for therapeutic application in inflammation-related conditions.

## 2. Materials and Methods

Acetonitrile (gradient grade for liquid chromatography) was obtained from Merck (Darmstadt, Germany), while formic acid was purchased from Sigma-Aldrich (Burlington, MA, USA). Curcumin reference standard was procured from ChemFaces (Wuhan, China). Absolute ethanol (analytical grade), methanol (HPLC grade), water (HPLC grade), glacial acetic acid, sodium nitrite, aluminum chloride, ferric chloride, ferrous sulfate, potassium persulfate, L-ascorbic acid, gallic acid, and quercetin were purchased from Daejung Chemical Co. (Busan, Republic of Korea). DPPH, ABTS, and sodium acetate were supplied by Sisco Research Laboratories (Mumbai, India). Folin–Ciocalteu phenol reagent and gallic acid were sourced from Sigma-Aldrich (St. Louis, MO, USA). Trolox^®^ and MTT reagent (3-(4,5-dimethylthiazol-2-yl)-2,5-diphenyltetrazolium bromide) were purchased from Thermo Scientific Chemicals (Waltham, MA, USA). Dulbecco’s Modified Eagle Medium (DMEM), 0.25% trypsin-EDTA, fetal bovine serum (FBS), and penicillin–streptomycin (P/S) solution were purchased from Gibco (Waltham, MA, USA). PGE-2 ELISA kit was purchased from R&D Systems (Minneapolis, MN, USA). The ELISA kits for the quantification of interleukin-1 beta (IL-1β), interleukin-6 (IL-6), and tumor necrosis factor-alpha (TNF-α) were purchased from ABclonal (Woburn, MA, USA). N^G^-Nitro-L-arginine methyl ester (L-NAME) and lipopolysaccharide (LPS) from Lipopolysaccharides from *Salmonella enterica* were purchased from Sigma-Aldrich (St. Louis, MO, USA). BAY 11-7082, a selective NF-κB pathway inhibitor, was obtained from MySkinRecipes (Bangkok, Thailand).

### 2.1. Plant Material Identification

The plant materials used in the Hand and Foot Soaking Formulary were collected from various regions across Thailand, as detailed in Table 1. A total of ten plant species were included, each identified and documented with a unique Thai Traditional Medicine (TTM) voucher number and collector information. The specimens were primarily collected by Dr. Nopparut Toolmal, and represent diverse floristic regions, including Northern, South–Eastern, Peninsular, Eastern, Central, and North–Eastern Thailand. The herbal materials utilized in this study were taxonomically identified by a certified plant taxonomist, Dr. Nopparut Toolmal, Department of Thai Traditional and Alternative Medicine, Ministry of Public Health, Thailand. Voucher specimens for each plant species were preserved at the Thai Traditional Medicine Herbarium in Bangkok, Thailand. The choice of specific plant parts for the Hand and Foot Soaking Formulary was informed by the National Thai Traditional Medicine Formulary, issued by the Ministry of Public Health, Thailand.

### 2.2. Hand and Foot Soaking Formulary Composition and Preparation

The Thai Traditional Hand and Foot Soaking Formulary consists of a blend of ten medicinal plant ingredients, each contributing specific parts known for their therapeutic properties (Table 2). To prepare the Hand and Foot Soaking Formulary, all dried plant materials were first milled into a fine powder to ensure homogeneity and facilitate effective extraction during use. The powdered herbs were accurately weighed according to the standardized formulation and thoroughly mixed to form a uniform blend. The resulting herbal mixture was then packed into heat-resistant filter paper sachets, each containing 30 g of the formulation.

### 2.3. Determination of Total Phenolic Content of the Hand and Foot Soaking Formulary

The total phenolic content (TPC) of the Hand and Foot Soaking Formulary and turmeric extract was determined using the Folin–Ciocalteu colorimetric assay, as described by Angsusing et al. [14]. Gallic acid solutions ranging from 1.95 to 1000 µg/mL were prepared to generate a standard calibration curve. The test samples were evaluated at concentrations of 10–5000 µg/mL. In a 96-well microplate, 100 µL of 10% (*v*/*v*) Folin–Ciocalteu reagent was added to each sample well, followed by 50 µL of 7.5% (*w*/*v*) sodium carbonate solution. The reaction mixtures were incubated at room temperature for 2 h in the absence of light. Absorbance was measured at 750 nm using a SpectraMax M3 multi-mode microplate reader (Molecular Devices, San Jose, CA, USA). The TPC was calculated from the gallic acid standard curve and expressed as micrograms of gallic acid equivalents per micrograms of dried crude extract (µg GAE/µg extract).

### 2.4. Determination of Total Flavonoid Content of the Hand and Foot Soaking Formulary

The total flavonoid content (TFC) of the Hand and Foot Soaking Formulary and turmeric extract was assessed using the aluminum chloride colorimetric method, as described by Angsusing et al. [14]. Quercetin was used as the reference standard at concentrations ranging from 1.95 to 1000 µg/mL. The test samples were prepared at a concentration of 10–5000 µg/mL. In each well of a 96-well plate, 100 µL of the sample was mixed with 30 µL of 5% (*w*/*v*) sodium nitrate and incubated for 5 min at room temperature. Next, 50 µL of 2% (*w*/*v*) aluminum chloride solution was added, and the mixture was incubated for 6 min. Finally, 50 µL of 1 N sodium hydroxide was added, and the reaction was allowed to proceed for 10 min. The absorbance was measured at 510 nm using a SpectraMax M3 multi-mode microplate reader (Molecular Devices, San Jose, CA, USA). The TFC was calculated based on the quercetin standard curve and expressed as micrograms of quercetin equivalents per microgram of dried crude extract (µg QE/µg extract).

### 2.5. Curcumin Quantification by HPLC

Curcumin quantification in the Hand and Foot Soaking Formulary and turmeric extract was conducted using an Agilent 1260 Infinity II HPLC system (Santa Clara, CA, USA), equipped with a quaternary pump, autosampler, multi-column thermostat, and photodiode array detector. The analytical method used for curcumin quantification was validated according to the AOAC International guidelines [15]. Chromatographic separation was achieved using an ACE 5 C18-AR analytical column (4.6 × 250 mm, 5 µm; Aberdeen, Scotland) coupled with a Phenomenex C18 guard column (4 × 3 mm, 5 µm; Torrance, CA, USA). The mobile phase comprised 0.1% formic acid in ultrapure water (phase A) and 0.1% formic acid in acetonitrile (phase B), delivered under isocratic conditions at 40:60 (A:B) for 12 min. The flow rate was maintained at 1 mL/min, the column temperature at 25 °C, and the detection wavelength at 420 nm. A 10 µL injection volume was used for all analyses. For standard curve generation, a stock solution of curcumin (200 µg/mL in ethanol) was serially diluted to yield working standards ranging from 8 to 40 µg/mL. Sample solutions were prepared by dissolving 50 mg of the crude extract of the Hand and Foot Soaking Formulary or turmeric extract in 50 mL of ethanol (1 mg/mL). All solutions were filtered through 0.45 µm nylon membrane filters prior to injection.

### 2.6. Antioxidant Assays

#### 2.6.1. DPPH Radical Scavenging Activity Assay

The DPPH radical scavenging activity of the test samples was evaluated following the method described by Tunit et al. [16]. A working DPPH solution was prepared by dissolving DPPH in methanol. Gallic acid and quercetin were used as a positive control at concentrations ranging from 2.15 to 1100 µg/mL. Curcumin as a bioactive compound in the Formulary was tested at concentrations of 1.95–1000 µg/mL. The extracts of the Hand and Foot Masking Formulary and turmeric were tested at concentrations ranging from 9.90 to 5066 µg/mL and 9.80 to 5017 µg/mL, respectively. In a 96-well plate, 100 µL of the DPPH solution was mixed with 100 µL of each sample. After incubation in the dark at room temperature for 30 min, absorbance was measured at 570 nm using a SpectraMax M3 multi-mode microplate reader (Molecular Devices, San Jose, CA, USA). The radical scavenging activity was calculated using the following formula:

$$DPPH\;Scavenging\;activity\left(\%\right)=\frac{A-B}{A}\times 100\%$$

where A is the absorbance of the control (DPPH solution with solvent only), and B is the absorbance of the DPPH solution with the sample. The IC_50_ value was determined from a dose–response curve, representing the concentration required to inhibit 50% of the DPPH radicals.

#### 2.6.2. ABTS Radical Scavenging Activity Assay

The ABTS radical scavenging assay was performed as described by Tunit et al. [16]. ABTS solution (7.4 mM) and potassium persulfate (2.6 mM) were mixed and incubated in the dark at room temperature for 24 h to generate ABTS•^+^ radicals. The solution was diluted with ethanol to adjust the absorbance to 0.7 ± 0.1 at 750 nm. In a 96-well plate, 5 µL of each test sample, including gallic acid (4.49–1150 µg/mL), quercetin (39.58–5066 µg/mL), curcumin (9.81–5025 µg/mL), Hand and Foot Masking Formulary (9.90–5066 µg/mL), and turmeric extracts (9.80–5016 µg/mL), was mixed with 145 µL of ABTS•^+^ solution. The mixture was incubated at room temperature in the dark for 15 min. Absorbance was then recorded at 750 nm using a SpectraMax M3 multi-mode microplate reader (Molecular Devices, San Jose, CA, USA). The percent scavenging activity was calculated using the following formula:

$$ABTA\;Scavenging\;activity\left(\%\right)=\frac{A-B}{A}\times 100\%$$

where A is the absorbance of the control (ABTS solution with solvent only), and B is the absorbance of the sample. The IC_50_ value was determined from a dose–response curve, representing the concentration required to inhibit 50% of the ABTS radicals.

#### 2.6.3. Ferric-Reducing Antioxidant Power (FRAP) Assay

The FRAP assay was carried out according to Tunit et al. [16]. The FRAP reagent was freshly prepared by mixing 10 mM TPTZ in 40 mM HCl, 20 mM FeCl_3_·6H_2_O, and 300 mM acetate buffer (pH 3.6), in a ratio of 10:1:1 (*v*/*v*). In each well of a 96-well plate, 180 µL of the FRAP reagent was added to 20 µL of the test sample. The tested substances included extracts of the Hand and Foot Soaking Formulary (9.90–5066 µg/mL), curcumin (2.25–1150 µg/mL), and turmeric extracts (9.80–5017 µg/mL), as well as standard antioxidants, gallic acid (2.15–1100 µg/mL) and quercetin (2.05–1050 µg/mL). After incubation at 37 °C for 30 min, absorbance was measured at 570 nm using a SpectraMax M3 multi-mode microplate reader (Molecular Devices, San Jose, CA, USA). The antioxidant capacity was calculated using a ferrous sulfate standard curve (9.8–2500 µM) and expressed as µmol Fe(II) equivalents per gram of dried extract.

### 2.7. In Vitro Cytotoxicity

#### 2.7.1. Cell Culture

RAW264.7 murine macrophage cells obtained from the American Type Culture Collection (ATCC; Manassas, VA, USA) (catalog number TIB-71) were cultured in DMEM supplemented with 10% FBS, 2 mM L-glutamine, and P/S solution. Cells were maintained in a humidified incubator at 37 °C with 5% CO_2_.

#### 2.7.2. Measurement of Cell Viability by MTT Assay

The cytotoxicity of the test samples was evaluated using the MTT assay as described by Chittasupho et al. [17]. RAW264.7 cells were harvested and seeded in 96-well plates at a density of 2 × 10^3^ cells/well. After 24 h of adherence, cells were treated with various concentrations (1.95 to 250 µg/mL) of the Hand and Foot Soaking Formulary and turmeric extract and 1.95–62.5 µg/mL of curcumin prepared in serum-free DMEM. Following 24 h of incubation at 37 °C in a 5% CO_2_ atmosphere, the medium was discarded, and 100 µL of fresh DMEM supplemented with 10% FBS and 0.5 mg/mL MTT solution was added to each well. Plates were incubated for an additional 3 h. The resulting formazan crystals were solubilized in 100 µL of DMSO, and absorbance was measured at 550 nm using a SpectraMax M3 multi-mode microplate reader (Molecular Devices, San Jose, CA, USA). Cell viability was calculated using the following equation:

$$\%\mathrm{Cell}\;\mathrm{Viability}=\frac{\mathrm{Absorbance}\;\mathrm{of}\;\mathrm{control}\;\mathrm{cells}}{\mathrm{Absorbance}\;\mathrm{of}\;\mathrm{trated}\;\mathrm{cells}}\times 100\%$$

### 2.8. Anti-Inflammatory Assays

#### 2.8.1. Nitric Oxide (NO) Production Assay

The inhibitory effect of the Hand and Foot Soaking Formulary, turmeric extract, and curcumin on nitric oxide production was assessed in LPS-stimulated RAW 264.7 murine macrophages using the Griess reagent method. Cells were seeded at 2 × 10^3^ cells/well in 96-well plates. Following 24-h incubation, cells were treated with 75 µL of the test samples at non-cytotoxic concentrations in serum-free DMEM and incubated for 3 h. Negative controls received 150 µL of serum-free medium, while positive controls received L-NAME (200 µM), an inhibitor of NOS.

After pretreatment, all wells were stimulated with 75 µL of LPS (10 µg/mL) to reach a final concentration of 5 µg/mL, and were incubated for an additional 24 h [18]. Then, 100 µL of the culture supernatant was transferred to a new plate for NO detection. Griess reagent was prepared by dissolving 250 mg sulfanilamide in 25 mL of 5% phosphoric acid, and 25 mg NED in 25 mL of deionized water. To each sample well, 50 µL of sulfanilamide and 50 µL of NED were added sequentially. The plate was incubated at room temperature in the dark for 10 min, and absorbance was measured at 540 nm using a SpectraMax M3 multi-mode microplate reader (Molecular Devices, San Jose, CA, USA). Sodium nitrite standards (0.59–300 µM) were used to construct a calibration curve, and the NO release was expressed as a percentage of the control group (LPS-treated group).

#### 2.8.2. Quantification of PGE_2_, TNF-α, IL-1β, and IL-6 Levels by ELISA

To evaluate cytokine inhibition, RAW 264.7 cells were seeded at a density of 2 × 10^3^ cells/well in 96-well plates and incubated for 24 h. After removing the spent medium, 100 µL of serum-free DMEM containing the test samples, vehicle control, or indomethacin (10 µM) was added. The cells were incubated for 3 h at 37 °C with 5% CO_2_. Negative controls received serum-free medium only, and positive controls were treated with 10 µM indomethacin or an NF-kB inhibitor BAY 11-7082 (10 µM) for cytokine suppression. Inflammatory stimulation was induced by adding 100 µL of LPS (final concentration 5 µg/mL) [19]. Plates were further incubated for 24 h. After incubation, culture media were harvested and analyzed for PGE_2_, TNF-α, IL-1β, and IL-6 secreted levels using ELISA kits, following the manufacturer’s protocol.

### 2.9. Statistical Analysis

All data were expressed as mean ± standard deviation (SD) from at least three independent experiments. Statistical analysis was conducted using GraphPad Prism version 8.0 (GraphPad Software, San Diego, CA, USA). One-way ANOVA followed by Tukey’s post hoc test was used for multiple group comparisons, while unpaired t-tests were used for two-group comparisons. A *p*-value of <0.05 was considered statistically significant.

## 3. Results

### 3.1. Evaluation of Total Phenolic Content in Hand and Foot Soaking Formulary

**Figure 1:**
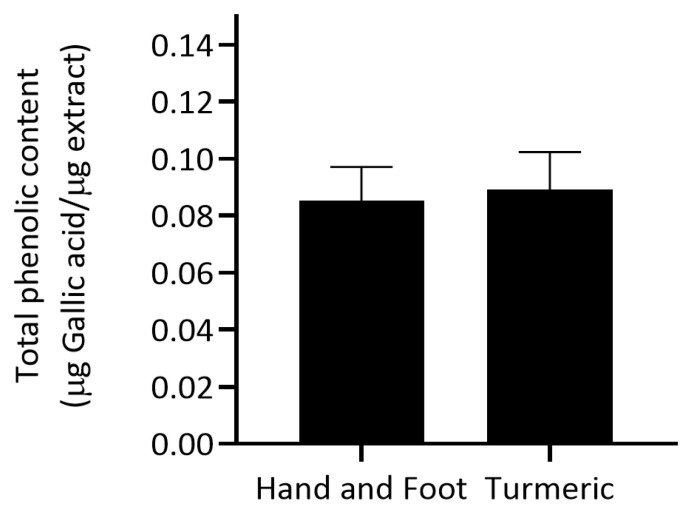
Total phenolic content of Hand and Foot Soaking Formulary and turmeric extract, measured as µg gallic acid equivalent per µg extract (µg GAE/µg extract). Data represent the mean ± standard deviation (*n* = 3).

The total phenolic content (TPC) of Hand and Foot Soaking Formulary and Turmeric extract was analyzed and expressed in micrograms of gallic acid equivalent per micrograms of extract (µg GAE/µg extract). The turmeric extract showed a slightly higher mean TPC (0.089 ± 0.013 µg GAE/µg extract) compared to the Hand and Foot Soaking Formulary (0.085 ± 0.012 µg GAE/µg extract). Both formulations demonstrated comparable levels of TPC, with turmeric extract exhibiting a marginally higher value than the Hand and Foot Soaking Formulary (Figure 1). Although turmeric extract exhibited a marginally higher TPC, the difference was not statistically significant (*p* > 0.05). This slight difference may be attributed to the inherent richness of turmeric in curcuminoids and other phenolic constituents known for their antioxidant and anti-inflammatory properties [20]. The relatively close TPC values suggest that the Hand and Foot Soaking Formulary may also contain significant contributions from phenolic-rich ingredients.

### 3.2. Total Flavonoid Content of Hand and Foot Soaking Formulary

**Figure 2:**
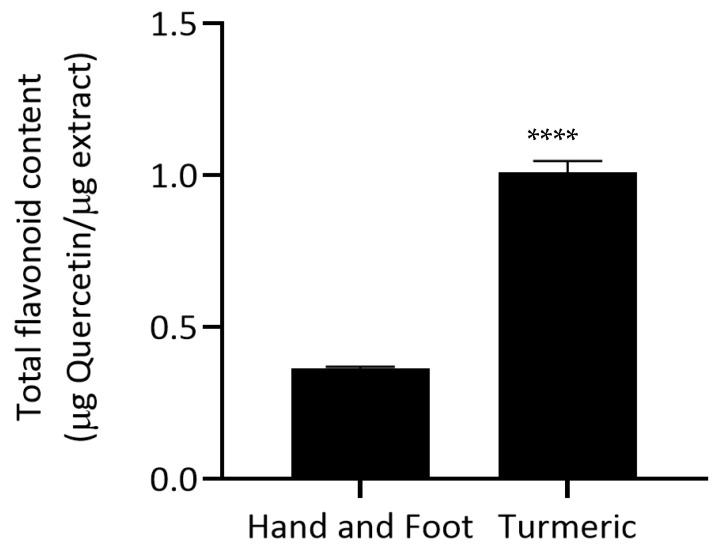
Total flavonoid content of Hand and Foot Soaking Formulary and turmeric extract, expressed as µg quercetin equivalent per µg extract (µg quercetin/µg extract). Values represent mean ± standard deviation (*n* = 3). **** *p* < 0.0001 compared to Hand and Foot Soaking Formulary.

The total flavonoid content (TFC) of the Hand and Foot Soaking Formulary and turmeric extract was quantified and expressed as micrograms of quercetin equivalent per microgram of extract (µg quercetin/µg extract). The turmeric extract showed a significantly higher flavonoid content (1.008 ± 0.038 µg quercetin/µg extract) compared to the Hand and Foot Soaking Formulary (0.363 ± 0.005 µg quercetin/µg extract), as shown in Figure 2, consistent with turmeric’s well-known richness in curcuminoids and other polyphenolic compounds [21].

### 3.3. Quantitative Determination of Curcumin Content in Hand and Foot Soaking Formulary

**Figure 3:**
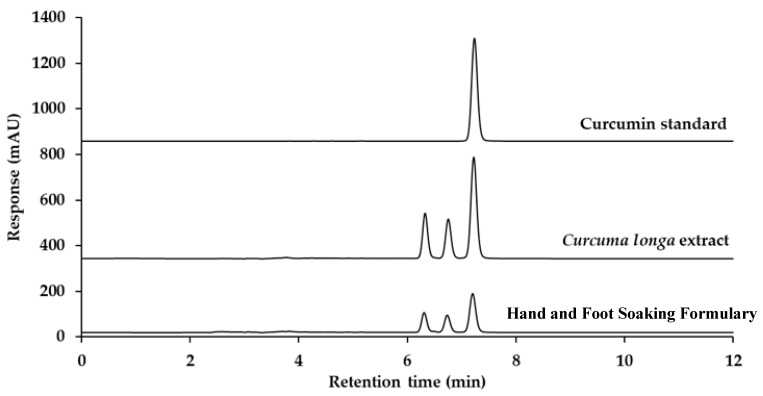
Representative HPLC chromatograms of the curcumin standard, turmeric (*C. longa*) extract, and the Hand and Foot Soaking Formulary.

The curcumin content in both the crude extract of turmeric and the Hand and Foot Soaking Formulary was determined using an HPLC analysis (Figure 3). A calibration curve was constructed using curcumin standard solutions, yielding a linear regression equation of y = 90.179x − 5.9557 with a correlation coefficient (R^2^) of 0.9999, indicating ex-cellent linearity. The analysis revealed that the crude extract of turmeric contained 63.01 ± 0.91 mg/g of curcumin with a %RSD of 1.45, which was slightly higher than the Hand Foot Soaking formulary’s curcumin content of 49.13 ± 1.12 mg/g, with a %RSD of 2.28. The curcumin content among the tested samples showed minimal variation, with %RSD val-ues below 5%, indicating consistent formulation without statistically significant differences. These results indicate that curcumin is retained at a substantial level during the formulation process, thus, the potential role of curcuminoids in the antioxidant and anti-inflammatory activities are further investigated in the following section.

### 3.4. DPPH Radical Scavenging Activity of Hand and Foot Soaking Formulary

**Figure 4:**
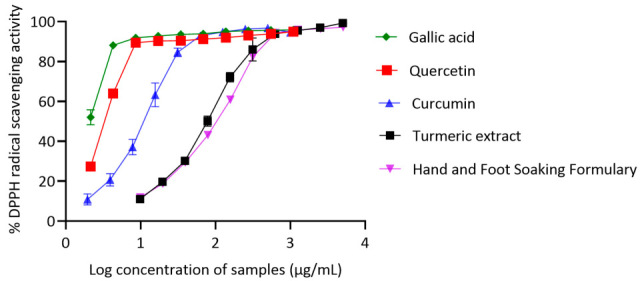
DPPH radical scavenging activity of gallic acid, quercetin, curcumin, turmeric extract, and the Hand and Foot Soaking Formulary. Results are expressed as mean ± SD (*n* = 3).

The antioxidant activity of the Hand and Foot Soaking Formulary and reference compounds, i.e., quercetin, gallic acid, curcumin, and turmeric extract, was determined using the DPPH radical scavenging assay. The results are shown in Figure 4. The IC_50_ values were calculated to compare their scavenging efficiencies. Quercetin and gallic acid exhibited the strongest antioxidant activity, with IC_50_ values of 3.19 ± 0.10 µg/mL and 1.93 ± 0.14 µg/mL, respectively. Curcumin showed moderate activity, with an IC_50_ of 14.46 ± 0.19 µg/mL, while turmeric extract displayed considerably weaker scavenging ability, with an IC_50_ of 65.25 ± 1.65 µg/mL. This reduced the activity of turmeric extract compared to pure curcumin, likely to be attributed to the dilution of active compounds or the presence of non-antioxidant constituents that can interfere with the assay [22]. The Hand and Foot Soaking Formulary showed a dose-dependent increase in DPPH radical scavenging, with an IC_50_ value of 107.93 ± 2.41 µg/mL.

The results of the DPPH assay indicate that quercetin and gallic acid are potent radical scavengers, requiring only low concentrations to exert their antioxidant effects. Curcumin, a polyphenolic compound from turmeric, also demonstrated moderate antioxidant activity, although its IC_50_ was higher than those of quercetin and gallic acid. This result corresponds to its known radical scavenging potential. In contrast, the turmeric extract exhibited substantially reduced antioxidant activity compared to pure curcumin, which could be attributed to the dilution of active compounds or the presence of non-antioxidant constituents in the crude extract. The Hand and Foot Soaking Formulary showed the antioxidant activity, with an IC_50_ of 90.41 µg/mL. This lower potency may be due to the presence of various constituents with mild or no radical scavenging capacity, or matrix interactions that hinder antioxidant function. Despite its higher IC_50_ value, the Formulary still demonstrated a clear dose–response relationship, suggesting the presence of bioactive phytochemicals that contribute to the overall antioxidant potential. While pure compounds like curcumin and gallic acid predictably showed strong radical scavenging activity, the performance of the Hand and Foot Soaking Formulary highlights the effectiveness of combining traditional herbal ingredients for functional antioxidant activity.

### 3.5. Comparative ABTS Radical Scavenging Activity of Curcumin, Turmeric Extract, and Hand and Foot Soaking Formulary

The ABTS radical scavenging activity of the Hand and Foot Soaking Formulary and selected reference compounds was evaluated and expressed as percentage inhibition at various concentrations, from which IC_50_ values were calculated. Gallic acid demonstrated the most potent ABTS scavenging activity, with an IC_50_ of 10.95 µg/mL, followed by curcumin (78.24 µg/mL) and quercetin (49.86 µg/mL). Turmeric extract showed moderate activity, with an IC_50_ of 322.6 µg/mL, whereas the Hand and Foot Soaking Formulary had the weakest ABTS scavenging effect with an IC_50_ of 622.5 µg/mL.

**Figure 5:**
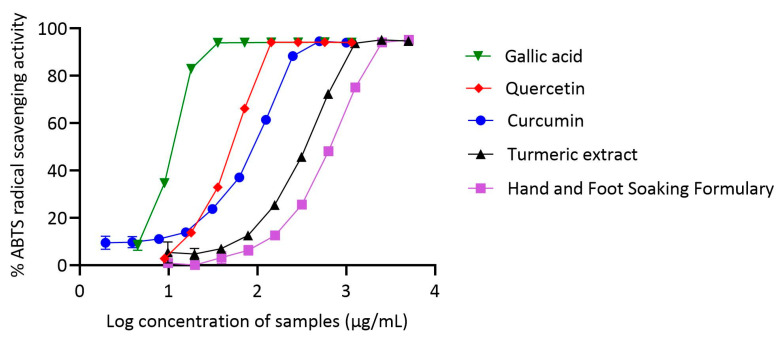
ABTS radical scavenging activity of gallic acid, quercetin, curcumin, turmeric extract, and the Hand and Foot Soaking Formulary. Data are presented as mean ± SD (*n* = 3).

Gallic acid exhibited exceptional activity, consistent with its known phenolic structure and high electron-donating ability. Curcumin also showed substantial antioxidant capacity, outperforming both quercetin and turmeric. Turmeric extract had moderate scavenging activity, but it was significantly lower than curcumin, likely due to the presence of non-antioxidant constituents diluting the effective concentration of curcuminoids. The Hand and Foot Soaking Formulary exhibited the lowest ABTS scavenging ability, with an IC_50_ of 622.5 µg/mL. This result suggests that although the Formulary contains various plant-derived constituents, their combined antioxidant activity in the ABTS assay is modest. The complex matrix may include compounds with low or no antioxidant properties, or interactions between components may hinder the activity of more potent constituents. Nonetheless, a concentration-dependent increase in scavenging activity was observed, reaching over 94% inhibition at the highest concentrations tested, indicating potential cumulative effects, though further studies are required to determine the activity at pharmacologically relevant doses (Figure 5).

### 3.6. Ferric-Reducing Antioxidant Power of Hand and Foot Soaking Formulary

**Figure 6:**
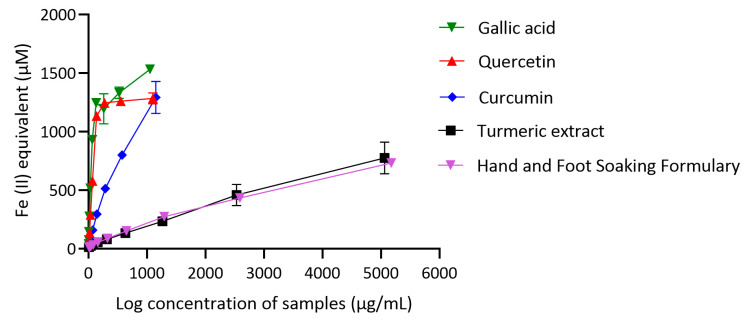
Ferric-reducing antioxidant power (FRAP) of gallic acid, quercetin, curcumin, turmeric extract, and the Hand and Foot Soaking Formulary. Data are presented as mean ± SD (*n* = 3).

The ferric-reducing antioxidant power (FRAP) of quercetin, gallic acid, curcumin, turmeric extract, and the Hand and Foot Soaking Formulary was evaluated at various concentrations. The reducing capacity increased in a dose-dependent manner for all tested samples. Among the standards, quercetin and gallic acid demonstrated the highest FRAP values (Figure 6). At 1100 µg/mL, quercetin exhibited a mean absorbance of 1284.86 ± 47.79, while gallic acid reached 1532.77 ± 17.93. Curcumin showed moderate reducing power, with a maximum value of 1292.38 ± 136.75 at 1150 µg/mL. For the herbal extracts, the Hand and Foot Soaking Formulary exhibited a concentration-dependent increase in FRAP activity, reaching 732.52 ± 110.88 at 5066.67 µg/mL. Turmeric extract displayed comparable activity, with a final value of 775.91 ± 134.99 at the same concentration.

The FRAP assay results confirm that quercetin and gallic acid are potent reducing agents, consistent with their known phenolic structures and strong electron-donating capabilities. These compounds readily reduce ferric ions to ferrous form, as evidenced by their high absorbance values even at relatively low concentrations. Curcumin also demonstrated strong reducing potential, although to a lesser extent than the reference phenolics. Its performance supports previous findings, indicating the redox-active nature of its β-diketone structure. The Hand and Foot Soaking Formulary and turmeric extract exhibited lower FRAP values and required much higher concentrations to achieve comparable reducing activity. The discrepancy between the extract and pure compounds concentration tested in this assay needs to be cautiously interpreted. Therefore, this outcome may reflect the complexity of their phytochemical profiles, which include several compounds with limited or no direct redox contribution. Nonetheless, both formulations showed a clear, dose-dependent increase in reducing power, suggesting the presence of functional antioxidants within their complex matrices. While their overall FRAP values were lower than the pure standards, the activity of the Formulary and turmeric extract supports their antioxidant capacity observed in other assays (DPPH and ABTS). These findings highlight the potential of multi-herbal preparations to exert antioxidant effects through multiple mechanisms, including radical scavenging and ferric ion reduction. Further identification of the active constituents within the Formulary may help explain its observed antioxidant capacity and support its traditional use.

### 3.7. Cytotoxicity Assessment of Hand and Foot Soaking Formulary in RAW264.7 Cells

**Figure 7:**
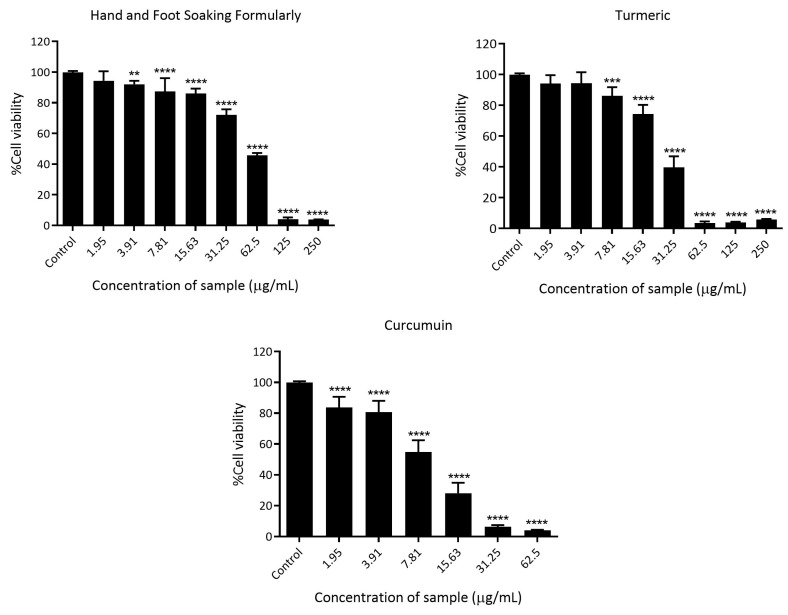
Percent cell viability of RAW264.7 cells treated with varying concentrations of Hand and Foot Soaking Formulary, turmeric extract, and curcumin as determined by MTT assay. Values represent the mean of three independent experiments. Data are presented as mean ± SD (*n* = 3). ** *p* < 0.01, *** *p* < 0.001, **** *p* < 0.0001 compared to vehicle control.

The cytotoxicity of the Hand and Foot Soaking Formulary, curcumin, and turmeric extract was evaluated in a dose-dependent manner (Figure 7). Cell viability decreased with increasing concentrations for all samples. At the lowest concentration tested (1.95 µg/mL), the Hand and Foot Soaking Formulary maintained high cell viability (94.38 ± 6.19%), similar to turmeric extract (94.19 ± 5.41%) and slightly higher than curcumin (83.80 ± 6.88%). However, as the concentrations increased, curcumin induced a more pronounced cytotoxic effect, reducing cell viability to 4.08 ± 0.27% at 0.0625 mg/mL and below 4% at higher concentrations. Turmeric and the Formulary retained higher viability at these doses.

The IC_50_ values (concentration causing 50% reduction in viability) were calculated as follows: curcumin showed the highest potency with an IC_50_ of 8.52 ± 1.65 µg/mL, followed by turmeric (27.63 ± 7.10 mg/mL) and the Hand and Foot Soaking Formulary (48.61 ± 3.80 mg/mL), indicating that the Formulary exhibited the lowest cytotoxicity among the tested samples. The analysis of dose–response curves, specifically comparing their HillSlopes derived from a four-parameter non-linear regression model, reveals distinct differences in how each compound elicits cytotoxicity. Curcumin displayed the steepest dose–response curve (HillSlope = 0.5331), indicating a more abrupt onset of cytotoxicity, even at lower concentrations. In contrast, both the Hand and Foot Soaking Formulary (HillSlope = 1.252) and turmeric extract (HillSlope = 0.9932) exhibited more gradual slopes. Statistical comparison of IC_50_ values and HillSlope parameters was performed using one-way ANOVA, followed by Tukey’s post hoc test. The differences between curcumin and both the Formulary and turmeric extract were statistically significant (*p* < 0.01), confirming that curcumin exhibits a significantly stronger and sharper cytotoxic effect. This suggests they induce a less abrupt cellular response and may have a broader effective concentration range. These findings imply that curcumin acts more potently and within a narrower concentration window, while the Formulary and turmeric extract exert their effects more gradually, possibly due to the buffered modulation of cytotoxicity by their complex mixtures of constituents.

The cytotoxicity profiles indicate that the Hand and Foot Soaking Formulary exerts minimal cytotoxic effects at lower to moderate concentrations compared to its active components. Curcumin, known for its bioactive properties, including anti-inflammatory and anticancer activities, demonstrated a strong cytotoxic effect with a low IC_50_ value, reflecting its potent activity on cellular viability. In contrast, turmeric extract showed a less potent effect, likely due to the presence of curcumin at lower concentrations, along with other constituents that dilute its activity. The Hand and Foot Soaking Formulary, a multi-herbal traditional preparation, showed the highest IC_50_ value, suggesting lower cytotoxicity. This may be due to a more balanced composition of phytochemicals that modulate biological activity without significantly affecting cell viability.

### 3.8. Effects of Hand and Foot Soaking Formulary, Turmeric Extract, and Curcumin on Lipopolysaccharide-Induced Nitric Oxide Production in RAW264.7 Macrophages

**Figure 8:**
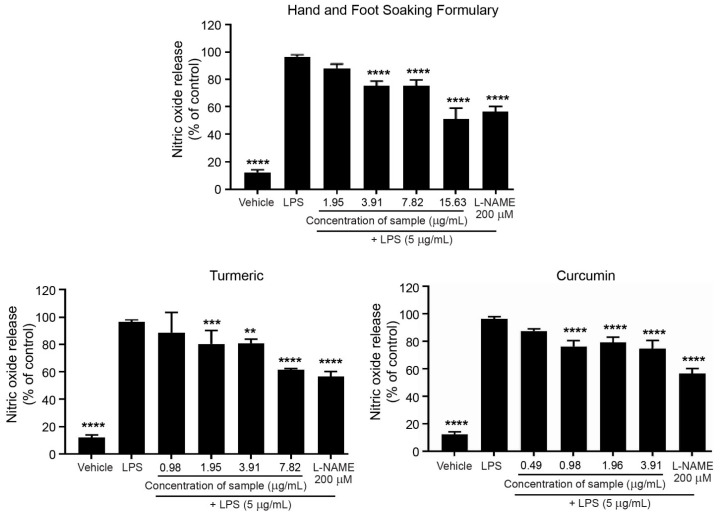
Effects of Hand and Foot Soaking Formulary, turmeric extract, and curcumin on nitric oxide (NO) release in LPS-stimulated RAW 264.7 macrophages. Data are presented as mean ± SD (*n* = 3). ** *p* < 0.01, *** *p* < 0.001, **** *p* < 0.0001 compared to LPS-treated control.

The inhibitory effects of the Hand and Foot Soaking Formulary, turmeric extract, and curcumin on nitric oxide (NO) production were evaluated in LPS-stimulated RAW264.7 macrophages (Figure 8). LPS treatment alone significantly increased NO levels to 96.31 ± 1.63%, compared to the untreated control (12.23 ± 1.93%), confirming successful induction of inflammation. As a positive control, L-NAME reduced NO production to 56.47 ± 3.82%, indicating significant inhibition of inducible nitric oxide synthase (iNOS) activity in RAW264.7 cells.

All tested samples demonstrated a concentration-dependent inhibitory effect of NO production. Curcumin exhibited the most potent NO inhibitory effect in a dose-dependent manner, with 71.73 ± 2.40% inhibition at 3.91 µg/mL. Turmeric extract also reduced NO production to 61.41 ± 1.04% at 7.815 µg/mL. The Hand and Foot Soaking Formulary showed modest inhibition of NO production to 50.91 ± 8.22% at 15.63 µg/mL, demonstrating dose-dependent activity. While these results confirm the potential of an anti-inflammatory effect in all tested samples, it is vital to distinguish the discrepancy of the concentration ranges used among each substance. Despite this, the formulation achieved the greatest reduction in NO production (up to 84.12% at 125 µg/mL). These differences highlight curcumin’s potency at lower doses, while also emphasizing the broader efficacy of the formulation, likely due to synergistic contributions from multiple herbal constituents.

The LPS-induced NO production assay in RAW264.7 macrophages is a well-established model for evaluating anti-inflammatory activity, particularly related to iNOS inhibitory action. In this study, curcumin showed the strongest potency per unit concentration for the NO-suppressive effect among the tested samples, consistent with its documented ability to downregulate iNOS tyrosine phosphorylation through inhibiting ERK 1/2 activation and subsequently suppressing iNOS enzyme activity [23]. When comparing IC_50_ values for NO inhibition and cytotoxicity, curcumin was the most potent inhibitor per unit concentration. Both turmeric extract and the Hand and Foot Soaking Formulary also showed substantial NO inhibition at a higher concentration. All three compounds exhibited favorable therapeutic indices. The effective anti-inflammatory concentrations were below cytotoxicity. Turmeric extract, although less potent than pure curcumin, still exerted significant inhibition, likely due to the presence of curcumin and other bioactive constituents contributing to the overall activity. Interestingly, the Hand and Foot Soaking Formulary also demonstrated greater NO inhibition in a concentration-dependent manner, albeit with lower potency compared to curcumin. The reduction in LPS-induced NO levels at higher concentrations suggests the presence of potential anti-inflammatory phytochemicals, possibly acting synergistically. While its effects were milder than pure compounds, the broader compositional matrix of the Formulary may offer balanced anti-inflammatory activity with lower cytotoxic risk, supporting its traditional use for inflammatory conditions.

### 3.9. Anti-Inflammatory Effects of Hand and Foot Soaking Formulary, Turmeric Extract, and Curcumin on LPS-Induced Inflammatory Cytokine Secretion in RAW264.7 Macrophages

LPS stimulation significantly increased the secretion of TNF-α, IL-6, and IL-1β in RAW264.7 macrophages compared to untreated controls. All tested samples, i.e., curcumin, turmeric extract, and the Hand and Foot Soaking Formulary, reduced cytokine levels in a concentration-dependent manner, with the formulation and turmeric extract showing more pronounced inhibition than curcumin at equivalent or higher concentrations. While direct mechanistic data were not collected in this study, previous reports have linked similar herbal components to the inhibition of NF-κB signaling and suppression of pro-inflammatory mediator expression [10]

**Figure 9:**
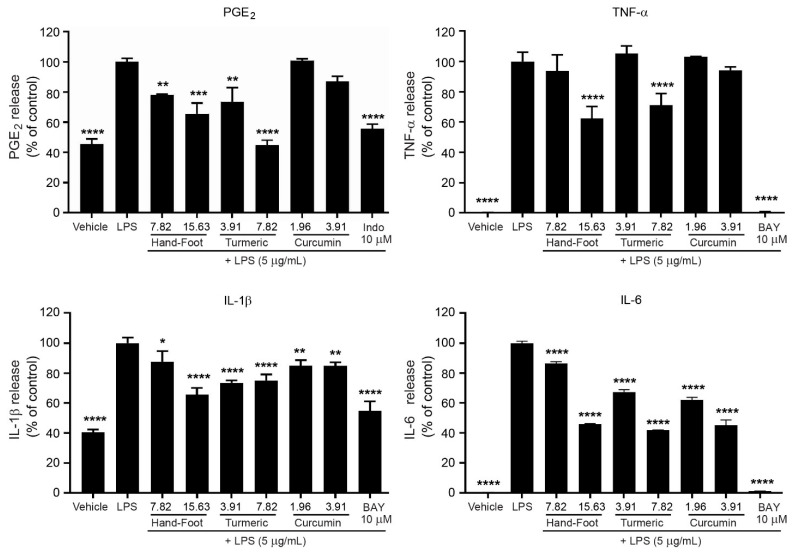
Effects of Hand and Foot Soaking Formulary, Turmeric extract, and Curcumin on pro-inflammatory cytokine and prostaglandin E_2_ (PGE_2_) production in LPS-stimulated RAW 264.7 macrophages. Data are expressed as % of LPS control and presented as mean ± SD (*n* = 3). * *p* < 0.05, ** *p* < 0.01, *** *p* < 0.001, **** *p* < 0.0001 versus LPS-treated group.

Treatment with LPS (5 µg/mL) significantly increased the release of inflammatory mediators, including PGE_2_, TNF-α, IL-1β, and IL-6, when compared to untreated control levels (Figure 9). Treatment with positive controls, indomethacin for PGE_2_, and BAY 11-7082 for cytokines, significantly suppressed these mediators, therefore validating the responsiveness of assays. All tested samples, including, Hand and Foot Soaking Formulary, turmeric extract, and curcumin, lowered pro-inflammatory cytokines in a concentration-dependent manner. In the PGE_2_ assay, the Hand and Foot Soaking Formulary reduced PGE_2_ to 65.57 ± 7.31% at the highest concentration, 15.63 µg/mL, while turmeric extract showed greater suppression, with a 44.60 ± 3.52% reduction at 7.82 µg/mL. Curcumin at 3.91 µg/mL showed less effects with 86.97 ± 3.50% inhibition.

A similar pattern was observed for TNF-α and IL-1β inhibition. At the highest concentration tested, the Hand and Foot Soaking Formulary reduced TNF-α to 62.39% and IL-1β to 63.20%, outperforming curcumin at its tested dose, with higher concentrations. IL-6 inhibition was the strongest for the Formulary at 45.93% and curcumin at 45.37%, followed closely by turmeric extract at 41.99%.

The Hand and Foot Soaking Formulary, turmeric extract, and curcumin demonstrated concentration-dependent inhibitory effects on pro-inflammatory mediators in LPS-stimulated models. This Formulary significantly reduced the release of PGE_2_, TNF-α, IL-1β, and IL-6 at higher concentrations, although to a lesser extent compared to specific inhibitors such as indomethacin and BAY.

Turmeric extract exhibited stronger LPS-induced PGE_2_ and IL-6 secretion than curcumin at the tested concentrations, suggesting the involvement of synergistic phytochemicals in turmeric extract beyond curcumin alone. Notably, curcumin showed potent inhibition of IL-6 and moderate reduction in TNF-α and IL-1β levels, consistent with its role in modulating NF-κB and MAPK signaling pathways.

The Hand and Foot Soaking Formulary displayed moderate yet consistent anti-inflammatory activity across all tested mediators. The multi-component nature of the Formulary likely contributes to its broad-spectrum efficacy, potentially acting through various inflammatory signaling routes, such as COX-PGE_2_, iNOS-NO, and NF-κB–cytokine axes. The data support its traditional use for alleviating inflammatory symptoms and indicate potential for further development as a natural anti-inflammatory formulation. However, proposed mechanisms remain speculative without direct mechanistic data. Although these interpretations are aligned with existing scientific data, including known activities of individual herbal ingredients, it should be noted that this should be further validated.

## 4. Discussion

Many recent studies demonstrate a strong positive correlation between the polyphenolic composition of plant extracts and their antioxidant and anti-inflammatory efficacy. In our study, the herbal extract possessed markedly high total phenolic content and total flavonoid content, along with antioxidant and anti-inflammatory activities. These results are consistent with reports in the literature that medicinal plants rich in phenolics and flavonoids tend to exhibit potent antioxidant and anti-inflammatory effects [24]. For example, a comparative analysis of 12 herbs showed that turmeric had the highest phenolic and flavonoid levels, and also expressed the strongest antioxidant activity among the group. Pearson’s correlation analysis in that study confirmed that TPC is a significant contributor to antioxidant capacity, with TFC also positively correlating to antioxidant activity [25]. Similarly, Hand and Foot Soaking Formulary extract contained high TPC/TFC values aligned with its effective bioactivity, suggesting that its phenolic constituents are major determinants of its antioxidant and anti-inflammatory potency. There are several proposed mechanisms of phenolics and flavonoids that contribute to antioxidant activities, such as directly neutralizing reactive oxygen species to form stable resonance-stabilized radical intermediates, metal chelation to prevent new radicals forming from metal catalysis, and activation of cytoprotective pathways [25]. Phenolics and flavonoids also exert significant anti-inflammatory effects through modulation of cellular signaling. Flavonoids suppress the NF-κB and MAPK signaling pathways, thus lowering the production of inflammatory mediators. As a result, cytokine levels such as TNF-α, IL-1β, IL-6 and IL-8, and inducible enzymes (COX-2 and iNOS) are downregulated in the presence of high phenolic content. Polyphenols also often stimulate AMP-activated protein kinase (AMPK) and Nrf2 pathways in tissues, which enhances both antioxidant enzyme levels and the anti-inflammatory state [26].

Collectively, these mechanisms illustrate how phenolic compounds could intercept inflammatory cascades at multiple points, including directly restraining the signaling pathways and enzymes that cause inflammatory responses. Our findings show that Hand and Foot Soaking Formulary contained considerable TPC and TFC, which are responsible for antioxidant and anti-inflammatory activities. In antioxidant assays, the Hand and Foot Soaking Formulary extract showed a high free radical scavenging capacity, which resulted from the ability to donate electrons or hydrogens to stabilize radicals. Hand and Foot Soaking Formulary extract exhibited anti-inflammatory effects by reducing pro-inflammatory cytokine release and inhibiting enzyme activity in cell-based assays. These effects align with the action of phenolic and flavonoid compounds for the NF-κB activation inhibition and help manage oxidative stress within tissues. The anti-inflammatory and antioxidant activity effects observed in Hand and Foot Soaking Formulary extract are likely due to the content of phenolic and flavonoid compounds. The relationship between phytochemical composition and biological activity supports the therapeutic potential. The results are also consistent with existing evidence that phenolic and flavonoid content are natural compounds that could be utilized for the treatment of oxidative stress and inflammation.

Lipopolysaccharide (LPS), known as a major factor involved in inflammation, is a component of the outer membrane of gram-negative bacteria, and is called endotoxin because of its endotoxic properties. LPS contributes to the pathogenesis of bacterial infection and can activate several immune cells, including macrophages [27]. LPS binds to the toll-like receptor 4 (TLR4) on macrophages, activating the canonical NF-κB signaling pathway [28]. NF-κB, a transcription factor complex, is responsible for many inflammatory genes, resulting in the production of inflammatory cytokines TNF-α, IL-6, IL-1β, nitric oxide (NO), and prostaglandin E_2_ (PGE_2_) [29]. In this study, LPS was used to induce inflammation in RAW 264.7 cells.

The Hand and Foot Soaking Formulary polyherbal extract demonstrated anti-inflammatory properties by lowering pro-inflammatory cytokines, namely, TNF-α, IL-1β, IL-6, NO, and PGE_2_ in LPS-stimulated RAW264.7 cells. These effects are linked to the inhibition of the NF-κB signaling pathway, as well as the suppression of iNOS and COX-2 activities from individual plant components. This study emphasizes turmeric (*C*. *longa*) and its principal bioactive compound, curcumin, despite the formulation containing ten different herbs. This focus is scientifically justified by the major component of turmeric that is present in the polyherbal mixture and the prominence of curcumin as a key pharmacological marker and active constituent of turmeric. It is likely that overall therapeutic effects were contributed by turmeric, since turmeric is a major ingredient in the formulary. In addition, the main active compound of turmeric, curcumin, is widely known for its antioxidant and anti-inflammatory properties. Curcumin blocks the activation of the NF-κB signaling pathway and reduces the expression of COX-2 and iNOS [30]. For completeness, it should be acknowledged that the other nine herbs in the formulation likely play complementary or synergistic roles in the overall therapeutic effect. This holistic approach is commonly found in Thai Traditional Medicine, contributing to the potential of synergistic or complementary effects among ten different herbal ingredients, which may differ from the action of isolated compounds. Although their individual contributions were not examined in this study, traditional polyherbal formulations often rely on supportive interactions among ingredients. Future investigations may explore individual ingredients in the formulary to determine their specific bioactivities and any synergistic effects. However, scientific evidence showed that each ingredient possesses anti-inflammatory properties. Piperine, from *P. retrofractum*, suppresses NF-κB activation and reduces TNF-α, IL-1β, and IL-6 release [31]. Citral, luteolin, and chlorogenic acid from *C. citratus* suppress NF-κB activation, downregulate iNOS, COX-2 expression, and lower IL-1β, IL-6, NO, and PGE_2_ in LPS-stimulated cells [32]. *C. micracantha*, which contains Pauciflorol E, reduced NO production via inhibition of iNOS expression [33]. Verbascoside, hispidulin, and rosmarinic acid, found in *C. indicum*, also inhibited NF-κB activation and suppressed iNOS, COX-2, and cytokine expression [34]. In addition to the inhibition of NO production, Limonoids in *H. perforata* suppressed both NF-κB and MAPK pathways [35]. *T. triandra* contains tiliacorinine and other alkaloids that could suppress NF-κB activation and reduce pro-inflammatory cytokines, including TNF-α, IL-1β, IL-6, and NO [36]. Several phytochemicals, such as triterpenoids, flavonoids, and tannins in *F. racemosa*, inhibit COX and LOX enzymes. Lupeol also suppressed NF-κB activation and reduced iNOS and COX-2 expression [37]. Flavonoids such as naringin and neohesperidin found in *C. hystrix* inhibited NF-κB and the NLRP3 inflammasome [38]; camphor and borneol found in the essential oil of *C. camphora* have the ability to downregulate inflammatory cytokine expression by modulating NF-κB and MAPK pathways [39]. This evidence highlighted the broad therapeutic potential of the Hand and Foot Soaking Formulary, likely due to the diverse ingredients acting together to produce the observed effect.

Nitric oxide (NO) is a free radical signaling molecule playing a role in many biological processes, including inflammation. NO is synthesized from L-arginine and oxygen (O_2_) and catalyzed by nitric oxide synthase (NOS). There are three main NOS isoforms, as follows: endothelial NOS (eNOS), neuronal NOS (nNOS), and inducible NOS (iNOS). The iNOS enzyme is a major isoform in the inflammatory process [40]. Thus, upregulation of iNOS might be reflected in NO production in the cells during inflammation. NO is immediately oxidized to nitrate and nitrite, which are both used to measure NO levels in the cells. LPS stimulation triggers the upregulation of iNOS in macrophages. To counteract the NO roles, L-NAME, a non-selective NOS inhibitor, is often employed [41]. Therefore, this study investigated the effects of the ethanol extract of Hand and Foot Soaking Formulary on the inhibition of LPS-mediated NO production by measurement of nitrate and nitrite levels in RAW 264.7 cells. NF-κB is one of the major regulators of inflammatory gene expression. The activation of NF-κB resulted in the upregulation of cytokines such as TNF-α, IL-1β, IL-6, and IL-8, including COX-2 [42].

LPS also triggers the COX-2 pathway, leading to PGE_2_ elevation. COX-2 converts arachidonic acid to prostaglandins that promote vasodilation, fever, pain, and inflammation. LPS exposure induces COX-2 expression in macrophages, resulting in increased PGE_2_ secretion [29]. Nonsteroidal anti-inflammatory drugs (NSAID), such as indomethacin, are commonly known as a non-selective inhibitor for the COX enzyme, including prostaglandin synthesis [43]. The study by Lin et al. revealed that indomethacin treatment inhibited LPS-induced PGE_2_ release in RAW264.7 cells. As a result, indomethacin mitigates the inflammatory effects of PGE_2_, notably via the COX-2 and iNOS pathways [43]. In our study, the Hand and Foot Soaking formulary, including turmeric extract and curcumin, suppressed PGE_2_ levels comparably to indomethacin, implying that the tested substances reduced prostanoid production through the COX-2 pathway. This likely occurs by downregulation of COX-2 expression.

Inflammation caused by LPS activation via NF-κB in macrophages produces pro-inflammatory cytokines, including TNF-α, IL-6, and IL-1β [28]. In response to LPS, NF-κB regulates the transcription of TNF-α, IL-6, and IL-1β. BAY 11-7082 is an inhibitor of IκB kinase that prevents NF-κB activation, which reduces LPS-induced TNF-α, IL-6, and IL-1β expression. The experimental model performed by Irrera et al. showed that LPS markedly increased TNF-α, IL-6, and IL-1β levels. After employing BAY 11-7082, the pro-inflammatory cytokines were lowered, as BAY 11-7082 blocks the nuclear translocation of NF-κB and significantly reduces LPS-induced TNF-α, IL-6, and IL-1β expression. We observed a similar reduction in these cytokines with our herbal extract, suggesting that NF-κB activity was diminished. LPS provides an NF-κB-driven priming signal for pro-IL-1β synthesis, which typically requires a second stimulus (e.g., NLRP3 inflammasome activation) for maturation and release. The fact that the Hand and Foot Soaking Formulary lowered IL-1β levels in an LPS-only model implies it interfered with this priming step, likely by inhibiting NF–κB-mediated IL-1β gene upregulation. Overall, the parallel between BAY 11-7082 and the herbal extract in dampening cytokine levels points to NF-κB inhibition as a key mechanistic node [44].

In our study, the Hand and Foot Soaking Formulary broadly suppressed inflammatory mediators, including NO, PGE_2_, TNF-α, IL-6, and IL-1β, in an LPS-stimulated macrophage model. These results suggest a multi-target mechanism, which could be explained by comparing it to the reference inhibitors employed in the experiment, L-NAME, indomethacin, and BAY 11-7082. However, rather than directly inhibiting each effector enzyme, the Hand and Foot Soaking Formulary acts by downregulating the expression or activity of upstream signaling proteins (like NF-κB or mitogen-activated kinases) that coordinate the inflammatory response. Our findings align with numerous reports on anti-inflammatory phytochemicals. Yun et al. [45] study indicated that asiatic acid, a triterpenoid from *Centella asiatica*, was shown to significantly inhibit LPS-induced iNOS and COX-2 expression, thus reducing NO and PGE_2_ release, and decreasing IL-1β, IL-6, and TNF-α levels by suppressing NF-κB activation. These broad actions mirror what we observed with the herbal extract, pointing to a likely NF-κB inhibitory effect. By preventing NF-κB activation, the extract would concurrently block the transcription of iNOS and COX-2, resulting in lowering NO and PGE_2_ production, as well as the transcription of pro-inflammatory cytokine genes. This mechanism is analogous to the action of BAY 11-7082, which targets the NF-κB signaling pathway, but is achieved with a natural mixture of bioactive compounds. In support of this, another recent study by Aeng et al. [46] on α-spinasterol, a phytosterol from *Achyranthes aspera*, demonstrated concomitant inhibition of LPS-induced TNF-α, IL-6, and PGE_2_; downregulation of COX-2; and a blockade of IKK/NF-κB signaling by that natural product. Such findings reinforce that the Hand and Foot Soaking Formulary in our study likely modulates the upstream signaling of TLR4–NF–κB and possibly MAPK pathways, rather than acting as a singular enzyme inhibitor. This gives it a multifaceted efficacy; in essence, the extract can comparably substitute for multiple specific inhibitors by targeting a higher-level control point in the inflammatory cascade.

The pro-inflammatory cytokines assessed in this study, including NO, PGE_2_, TNF-α, IL-1β, and IL-6, are commonly caused by skin inflammation. RAW264.7 macrophage cells were used as a validated model for inflammatory conditions. The inhibition of LPS-stimulated cells provided a mechanism relevant to topical applications. A previous study by You et al. [47] showed that the substances that inhibit pro-inflammatory markers in macrophage also attenuate skin inflammation in vivo. Given the traditional use of the Hand and Foot Soaking Formulary, these effects support its potential clinical efficacy in managing inflammatory skin conditions.

The in vitro antioxidant and anti-inflammatory activity of Hand and Foot Soaking formulary provide strong pre-clinical findings supporting its traditional application. The complexity of synergistic actions due to its diverse herbal components, rather than a single active substance, represents a key point of originality and a significant advantage for topical herbal remedy. The multiple actions may offer more comprehensive benefits and reduce side effects compared to individual compound treatment, complying with the holistic principle of Thai Traditional Medicine.

Although our findings provide solid in vitro results, it is important to acknowledge the limitation of this experiment. While RAW264.7 macrophage cells were used for a cell culture model for mechanistic investigation, they do not fully represent the complexity of the in vivo biological system, such as pharmacokinetics. Therefore, the interpretation of effects observed in our study should be further validated. Additionally, apart from curcumin, other components in the formulary should be further investigated and quantified for the complete phytochemical profile.

## 5. Conclusions

Thai Traditional Medicine is an ancient medical system that is attracting scientific interest for its skincare applications. Recent research, including in vitro studies, supports the effectiveness and safety of TTM topical treatments for conditions such as inflammation, infections, and aging, validating traditional practices. This study demonstrated the antioxidant and anti-inflammatory potential of a traditional Thai Hand and Foot Soaking Formulary and its active compound curcumin. The formulation showed notable phenolic and flavonoid contents and a high curcumin level. It exhibited strong radical scavenging activity, moderately reducing power in in vitro assays. Furthermore, cytotoxicity testing in RAW264.7 cells showed non-cytotoxic at concentrations below 15.63 µg/mL. In LPS-stimulated RAW264.7 macrophages, it effectively reduced nitric oxide production and pro-inflammatory cytokines, comparable to turmeric extract and curcumin. These findings support its potential as a safe and effective herbal product for topical applications targeting oxidative stress and inflammation by modulating key pathways such as iNOS, COX, and NF-κB. While these in vitro findings support its traditional use in Thai Traditional Medicine and suggest its promise as a natural product for topical application in alleviating inflammation and oxidative stress, further in vivo studies are essential to validate the topical application and confirm the clinical efficacy.

## Figures and Tables

**Table 1:** Plant Materials and Voucher Specimens Used in Hand and Foot Soaking Formulary.

Scientific Name	Family	Vernacular Name	Part Use	Collector	TTM	Source
*Curcuma longa* L.	Zingiberaceae	Khamin Chan	Rhizome	Toolmal et al. 190717014	0003794	San Tha subdistrict, Na Noi district, Nan province, Thailand
*Piper retrofractum* Vahl	Piperaceae	Di Pli	Fruit	Toolmal et al. 611153010	0000129	Makham subdistrict, Khlung district, Chanthaburi province, Thailand
*Cymbopogon citratus* (DC.) Stapf	Poaceae	Ta Khrai	Rhizome	Toolmal et al. 100517017	0003656	Wisai Nuea subdistrict, Mueang district, Chumphon province, Thailand
*Capparis micracantha* DC.	Capparaceae	Chingchi	Root	Toolmal et al. 071117002	0000376	Hua Na Kham subdistrict, Kranuan district, Khon Kaen province, Thailand
*Clerodendrum indicum* (L.) Kuntze	Lamiaceae	Thao Yai Mom	Root	Toolmal et al. 600354041	0003852	Klang subdistrict, Det Udom district, Ubon Ratchathani province, Thailand
*Harrisonia perforata* (Blanco) Merr.	Simaroubaceae	Khontha	Root	Toolmal et al. 600354029	0000349	Pluakdaeng subdistrict, Pluakdaeng district, Rayong province, Thailand
*Tiliacora triandra* (Colebr.) Diels	Menispermaceae	Thao Ya Nang	Root	Toolmal et al. 480254059	0000337	Pluakdaeng subdistrict, Pluakdaeng district, Rayong province, Thailand
*Ficus racemosa* L.	Moraceae	Ma Duea Utum Phon	Root	Toolmal et al. 261053063	0000279	Salaya subdistrict, Phutthamonthon district, Nakhon Pathom province, Thailand
*Citrus hystrix* DC.	Rutaceae	Makrut	Fruit (rind)	Toolmal et al. 520154113	0000066	Hua Na Kham subdistrict, Kranuan district, Khon Kaen province, Thailand
*Cinnamomum camphora* (L.) J.Presl (camphor)	Lauraceae	Karabun	Wood	Toolmal et al. 261054010	1000114	Bangkok province, Thailand

**Table 2:** Thai Traditional Hand and Foot Soaking Formulary Composition.

No.	Plant Scientific Name	Part Used	% *w*/*w*
1	*Curcuma longa* L.	Rhizome	20
2	*Piper retrofractum* Vahl	Fruit	10
3	*Cymbopogon citratus* (DC.) Stapf	Rhizome	10
4	*Capparis micracantha* DC.	Root	10
5	*Clerodendrum indicum* (L.) Kuntze	Root	10
6	*Harrisonia perforata* (Blanco) Merr.	Root	10
7	*Tiliacora triandra* (Colebr.) Diels	Root	10
8	*Ficus racemosa* L.	Root	10
9	*Citrus hystrix* DC.	Fruit (rind)	10
10	*Cinnamomum camphora* (L.) J.Presl	Wood	1

## Data Availability

Data are contained within the article and Supplementary Materials.

## Supplementary Materials

The following supporting information can be downloaded at: https://www.mdpi.com/article/10.3390/pharmaceutics17070907/s1, Table S1. DPPH Radical Scavenging Activity of Hand and Foot Soaking Formulary.
