# Supplementary material for: Antioxidant and Anti-Inflammatory Activities of Thai Traditional Hand and Foot Soaking Formulary and Its Bioactive Compounds
Source: Pharmaceutics. 2025 Jul 13;17(7):907. doi: 10.3390/pharmaceutics17070907 (PMC12298766; doi:10.3390/pharmaceutics17070907)
Supplement: Supplementary file 1 [file pharmaceutics-17-00907-s001.zip › pharmaceutics-3709283-supplementary.pdf]

Antioxidant and Anti-Inflammatory Activities of Thai Traditional Hand and Foot Soaking Formulary and Its Bioactive Compounds

Jaenjira Angsusing [1], Weerasak Samee [2], Supachoke Mangmool [3], Usma Dortae [1], Pranot Keawthip [1], Surakameth Mahasirimongkol [1], Somsak Kreechai [1], Kulthanit Wanaratna [1], Chuda Chittasupho [3]* and Nopparut Toolmal [1]*

[1] Department of Thai Traditional and Alternative Medicine, Ministry of Public Health, Nonthaburi 11000, Thailand (jaenjira.angsusing@hotmail.com, usma.do@dtam.mail.go.th, pranot.ka@dtam.mail.go.th, surakameth.m@dmsc.mail.go.th, somsak.kreechaii@gmail.com, kulthanitw@gmail.com, nopparut.to@dtam.mail.go.th)

[2] Faculty of Pharmacy, Srinakharinwirot University, Nakhon Nayok 26120, Thailand; (weerasak@g.swu.ac.th)

[3] Faculty of Pharmacy, Chiang Mai University, Chiang Mai 50200, Thailand; (supachoke.man@cmu.ac.th, chuda.c@cmu.ac.th)

* Correspondence: 1. Assoc Prof Dr. Chuda Chittasupho, Faculty of Pharmacy, Chiang Mai University, Chiang Mai 50200, Thailand, Email: chuda.c@cmu.ac.th, 2. Dr. Nopparut Toolmal, Department of Thai Traditional and Alternative Medicine, Ministry of Public Health, Nonthaburi 11000, Thailand, Email: nopparut.to@dtam.mail.go.th

Table S1. The IC_{50} values of DPPH Radical Scavenging Activity of Hand and Foot soaking Formulary

Table S1. DPPH Radical Scavenging Activity of Hand and Foot Soaking Formulary

Sample	IC_{50} (μg/mL)
Gallic acid	1.93±0.14
Quercetin	3.19±0.10
Curcumin	14.46±0.19
Turmeric extract	65.25±1.65
Hand and Foot Soaking Formulary	107.93±2.41
